# Clinical Training during the COVID-19 Pandemic: Experiences of Nursing Students and Implications for Education

**DOI:** 10.3390/ijerph19106352

**Published:** 2022-05-23

**Authors:** Magdalena Dziurka, Michał Machul, Patrycja Ozdoba, Anna Obuchowska, Michał Kotowski, Aleksandra Grzegorczyk, Aleksandra Pydyś, Beata Dobrowolska

**Affiliations:** 1 Students’ Scientific Association at the Department of Holistic Care and Nursing Management, Medical University of Lublin, 20-081 Lublin, Poland; pozdoba@op.pl (P.O.); annaobuchowska2@gmail.com (A.O.); komigato@gmail.com (M.K.); aleksandragrze.97@gmail.com (A.G.); olapydys@gmail.com (A.P.); 2 Department of Holistic Care and Nursing Management, Faculty of Health Sciences, Medical University of Lublin, 20-081 Lublin, Poland; michal.machul@umlub.pl (M.M.); beata.dobrowolska@umlub.pl (B.D.)

**Keywords:** clinical education, nursing students, qualitative research, COVID-19 pandemic, professional education

## Abstract

The COVID-19 pandemic has caused difficulties in the organization of clinical classes for nursing students. It is therefore important to explore students’ experiences related to participation in clinical classes during the pandemic and to draw conclusions that will allow for the introduction of innovations enabling the development of the required professional competencies as part of training during current and future pandemic restrictions. In this study, we aimed to explore the experiences of nursing students related to clinical education during the COVID-19 pandemic and to identify practical implications for this education in the future. A qualitative study was performed based on individual interviews among Polish nursing students (*n* = 20). The study is reported using the COREQ checklist. Content analysis was applied, and five main categories were identified, including ‘the key role of clinical mentor’, ‘theory-practice gap’, ‘ambivalent emotions and ethical challenges’, ‘to be part of the team’, and ‘strengthened professional identity’. The results of our research indicate that higher education institutions should implement clear strategies to support students, both in terms of psychological support and compensation of professional skills, the development of which might be limited during the pandemic. Modern technologies, including medical simulations, virtual reality, artificial intelligence, and telemedicine should be used in the practical teaching of nursing students to educate them on how to cope with difficult, new situations, build decision-making skills, and solve problems.

## 1. Introduction

The first information regarding the isolation of a new type of coronavirus appeared in January 2020 in the WHO situational report. At the turn of 2020, the Chinese WHO office was informed about cases of pneumonia of unknown etiology observed in the city of Wuhan in the Hubei province in China [1]. The rapid spread of the second coronavirus (severe acute respiratory syndrome, SARS-CoV-2, causing COVID-19) led to the classification of the disease as a pandemic on 11 March 2020 [2].

The state of emergency announced in relation to the pandemic necessitated the introduction of urgent changes in education, including transitioning from on-site to remote teaching with the use of distance-learning methods and techniques. Universities educating future nursing students have similarly responded to the clinical educational needs of nursing students at this unusual time, suspending in-person classes and relying on virtual clinical experiences [3,4]. These changes have affected more than 1.570 million students in 191 countries. They were associated with a global vision of challenges which involved the provision of adequate clinical experiences using simulations, virtual reality, and telemedicine [5]. These challenges have been taken up, among others, by the Society for Simulation in Healthcare, which developed a number of strategies, including how to employ high-fidelity simulations, and created a code of ethics for healthcare simulation workers [6].

Despite technological achievements, students have pointed out many difficulties in the practical application of theoretical knowledge in virtual settings and, in the case of university graduates, adaptation to working conditions and the hospital environment. They described the transition from formal education to nursing practice as stressful, especially during the pandemic [7].

The process of professional preparation of nurses at universities is quite burdensome, involving many hours of practical and theoretical classes, rigorous exams, and students’ concerns about their academic performance and postgraduate plans. These factors can trigger anxiety and even depressive symptoms [8,9]. Although anxiety was widespread among nursing students in the pre-pandemic years, it only intensified following the appearance of the SARS-CoV-2 virus. The literature reports several difficulties affecting students in connection with e-learning, from logistical problems due to insufficient electronic equipment or network inaccessibility to immature and adverse ways of coping with stress [10,11,12,13,14]. These are a consequence of modifications to the forms of teaching, changes in schedules and guidelines, difficulties in completing clinical placements, and concerns about potential delays in graduation and receipt of diplomas. Students’ mental state was also affected by the inability to predict the exact end to the pandemic and the loneliness resulting from isolation [11,15].

Furthermore, research conducted globally showed that nursing students who provided voluntary help during the COVID-19 pandemic experienced fear [16,17]. The educational process was described by the students as disorganized, requiring self-learning, lonely, and erratic; however, at the same time, as a unique learning opportunity [18]. Casafont et al. [19] showed that experiences of Spanish four-year nursing students as healthcare assistants during the COVID-19 pandemic provoked ambivalent emotions. Students assessed this experience as highly valuable in reference to the skills and knowledge they acquired. However, the lack of professional standards, guidelines, or protocols specifying methods of treatment of COVID-19 evoked emotions such as fear, anxiety, and sadness. In addition, the students learned how to adapt to new, unknown circumstances and thus felt proud and helpful.

In the face of sudden changes to the practical education of nursing students caused by the COVID-19 pandemic, it is important to understand the students’ experiences associated with participation in clinical classes and to draw conclusions that will enable the introduction of innovations that will in turn allow the best possible development of the required professional competences during the educational process, despite pandemic restrictions. The broadening of curricula to include more content on infectious diseases (control of infection or selection and proper use of personal protective measures), as suggested by the American Association of Colleges of Nursing, has been recognized as crucial to ensuring the safety of nursing students in the future [20]. This is one of the many directions of change following the experiences of nursing education during the pandemic, including greater use of modern technologies in nursing education, e.g., online classes [5,21,22], use of a game-based computer learning application that requires nursing students to solve problems through simulated cases [23,24], online virtual simulation [25], e-health solutions, and telenursing [26,27]. Therefore, the aim of this study was to explore the experiences of nursing students regarding clinical training which they attended as part of their professional education during the COVID-19 pandemic, and to identify practical implications for this education in the future.

## 2. Materials and Methods

### 2.1. Aim

The aim of this study is twofold: to explore the experiences of nursing students associated with clinical education during the COVID-19 pandemic, and to identify practical implications for this education in the future. Three research problems were stated: (1) What were the nursing students’ experiences associated with their relationship with patients, their families, clinical mentors, nursing staff, and peer groups during clinical training during the pandemic? (2) What facilitators and barriers did they experience during clinical training during the pandemic? Finally, (3) What practical implications are coming from students’ experiences that can aid in the development of clinical nursing education?

### 2.2. Study Design

Qualitative research using the structured interview method was carried out in eastern Poland between June and October 2021, i.e., more than a year after the first identified case of COVID-19 in Poland, and followed a year-long experience of training provided to nursing students during the pandemic. In order to comply with the prescribed standard by reducing the risk of bias and maximizing the accuracy and reliability of the study, the following steps were performed, as recommended by Johnson et al. [28]: (1) the goal of the study, research problems, and criteria for inclusion in the study were clearly and precisely specified on the basis of a thorough review of the available literature prior to the start of the study; (2) the sampling method (purposive sampling methodology) was selected on the basis of the conceptual framework of the study; (3) the interviews were analyzed by two researchers who did not participate in the collection of interviews to prevent subjective bias and ensure dependability, credibility, transferability, and confirmability of results; (4) the authors presented limitations of the study that may have affected its results; (5) determination of data saturation was performed; (6) the study was conducted in accordance with ethical principles after obtaining the consent of the bioethics committee; (7) the interviewer was not engaged in any dependent relationship with the interviewed students; and (8) the interviews and their transcription were conducted by the same researcher prepared to collect interviews, retaining a uniform manner of conversation and atmosphere, and the interviews were recorded and transcribed word-for-word immediately after their completion. Moreover, the study is reported using the COREQ (Consolidated criteria for Reporting Qualitative research) checklist (File S1), [29].

### 2.3. Study Participants and Setting

The study involved 20 undergraduate (BA) and graduate (MA) nursing students from eastern Poland. The study group comprised 17 women and 3 men with an average age of 22 years. The number of study participants was determined after data saturation had been reached during the collection of interviews (i.e., no additional and new information had been identified, and repetition of data has been recognized) and its rationale is provided in the literature on qualitative research [30,31]. The purposive sampling methodology was applied. The criteria for inclusion in the study were as follows: the status of an undergraduate (BA) nursing student (Years 2 and 3) or graduate (MA) nursing student (Year 1) with a record of clinical education during the COVID-19 pandemic and consent to participate in the study.

Nurses’ education in Poland is based on the educational standards of the Ministry of Education and Science [32,33,34]. Bachelor’s (BA, firstcycle) and master’s (MA, second-cycle) nursing studies can be distinguished; the exact organizational characteristics of the respective programs are provided in Table 1 [32,33,34].

Practical classes are held in simulation-based conditions to apply and deepen the acquired knowledge and professional skills. Professional internships are provided in order to help students master the professional skills that are fundamental for work in the nursing profession [33]. In the academic year 2020/2021, due to the COVID-19 pandemic, nursing students who performed assignments that were part of activities carried out by medical facilities or sanitary and epidemiological authorities in connection with SARS-CoV-2 virus infections (i.e., by working professionally as MA students or as volunteers) could apply for credit for professional internships, which included practical skills that they acquired when performing these activities. Nursing students could also obtain up to 20% of the ECTS (European Credit Transfer System) credits assigned to practical classes and professional internships [34] as part of classes conducted using distance-learning methods and techniques.

### 2.4. Research Instrument

Participants were asked eight basic questions which guided the whole interview. The questions are listed in Table 2. At the beginning, all participants were asked several questions regarding their socio-demographic characteristics. These questions were pilot tested with 4 participants. Data from interviews conducted in the pilot test were included in the final analysis.

### 2.5. Data Collection Process

Interviews were collected directly (*n* = 13) as well as (due to epidemiological restrictions) through telephone conversations (*n* = 7). Information about the study was prepared in the form of a graphic explaining the purpose and course of the study and providing the interviewer’s contact details. This information was posted in nursing student groups on social media and distributed via e-mail to the heads of BA and MA student groups with a request for dissemination in accordance with the inclusion criteria. Students willing to participate contacted the interviewer, and together they set a convenient date for the interview. Overall, 20 students applied and all of them took part in the study.

All interviews were recorded and transcribed verbatim using Microsoft Word 2017 software immediately after completion. Field notes were made by M.M. after every interview. These field notes were used to assist in the analysis of the transcribed audio recordings. Data collection and analysis took place in parallel, which allowed the main categories to be identified and the data collection process to be completed after data saturation was reached [30,31]. The conversations lasted between 14 and 23 min, with an average of 17 min. All interviews and transcriptions were conducted by the same male researcher (M.M.), a graduate of nursing (MA) and a doctoral student prepared to collect interviews for research purposes. Face-to-face (direct) conversations were conducted in an environment that ensured peace and quiet, with epidemiological recommendations taken into account. Similarly, telephone interviews took place at times arranged in advance to avoid haste and lack of concentration. Since interviews were conducted by the same researcher, the conversations and their atmosphere followed a uniform pattern. 

Each student was assigned a code (St. 1, St. 2, etc.) in order to ensure anonymity and facilitate identification of individual quotes during the analysis and presentation of the results.

### 2.6. Qualitative Analysis

The content of the interviews was analyzed by two researchers using the method proposed by Burnard [35]. This method of analysis is an interpretative process that focuses on the main topic and examines the similarities and differences between and within individual interviews. The two researchers, independently of each other, repeatedly listened to the participants’ recordings and read the transcripts of the interviews to fully understand the meaning of the participants’ accounts and descriptions of their clinical learning experiences during the pandemic. Content analysis was carried out according to the following stages: (a) identification of the main thematic categories by searching for similarities and thematic differences (the researchers noted words or phrases that summarized all of the data) (b) reviewing the identified categories (words and phrases were grouped together and those which were repeated or were synonyms were eliminated in order to select a final set of categories) and ensuring that all material collected in interviews had been classified into selected thematic categories; and (c) identification of student accounts (citations) with distinguishing features for a given category. The analyses carried out by two researchers were compared, and any ambiguities were resolved by checking the transcript and discussing the final wording of the thematic categories with the rest of the authors.

### 2.7. Ethical Issues

Participation in the study was voluntary. Information about the study was provided to the students before they gave their consent to participate. Confidentiality and anonymity were ensured by deleting all identifying information prior to the processing of any data. To ensure anonymity, the subjects were assigned encrypted codes. The researcher did not remain in any relationship of dependence with the respondents, i.e., he was not their teacher. The consent of the Bioethics Committee of the Medical University of Lublin No. KE-0254/289/2020 was obtained.

## 3. Results

### 3.1. Study Participants

Seventeen women (85%) and three men (15%) took part in the study. Their average age was 22 years old (SD = 1.19). The majority of students represented the 1st level of education, bachelor’s degree studies (*n* = 15; 75%). More than half of the respondents lived in urban areas (*n* = 12; 60%), (Table 3).

### 3.2. Identified Categories

Five main categories were identified: (1) the key role of a clinical mentor; (2) theory–practice gap; (3) ambivalent emotions and ethical challenges; (4) to be part of the team; (5) strengthened professional identity.

#### 3.2.1. The Key Role of a Clinical Mentor

The analysis of interviews revealed students’ conflicting feelings about their clinical teachers who worked as nurses and shared with them their knowledge, skills, and experience. Despite this, students recognized clinical mentors’ role in education during a period of national lockdown. They clearly underlined that despite the inability to conduct clinical training in the hospital during the first pandemic wave, mentors tried to teach them theoretical knowledge and practical skills at the highest level, seeking alternative methods of education, so that students in their future work could provide the highest quality of healthcare. Students also stressed that the mentors apparently found it important to protect students’ physical and mental health during their education, both at the university and in the hospital. Examples of students’ positive feedback about their clinical mentor include:

*“Some of them taught their classes better, others not so much, but almost all of them kept it at a certain level and provided us with practical knowledge as much as possible, even though the wards were closed and simulation rooms were inaccessible.”*

(St. 1)

*“Both mentors from the university and ward nurses protected us from coronavirus infection. Each of the lecturers paid special attention to keeping distance, wearing masks, and disinfecting.”*

(St. 2)

Furthermore, students observed that teachers were more supportive and more understanding in this difficult situation and whenever any problems appeared. Some students reflected that their contact with the mentor during clinical training was the same as before the pandemic. Students also appreciated good communication with the mentors as well as their patience and evident willingness to complete the didactic material, hence the good relations between the students and the mentors despite the stressful burden of the pandemic. The respondents also pointed out that due to the large number of classes, it took them more time than usual to master the applicable procedures and modes of conduct. Cooperation with the mentors and the fact they were willing to devote their time to revise and explain the material had a positive impact on the mutual relationships.

*“During all these internships, the staff members were in general very helpful and wanted to show us as many things as possible. They also let us perform most of the nursing activities and were happy to answer any questions.”*

(St. 1)

*“They carried out their responsibilities very well. They were very helpful throughout this period and made it easier for us to study, so that we would not be stressed too much with this situation; after all, it’s independent of us.”*

(St. 6)

However, some students felt abandoned and missed contact with their clinical mentors. Students also felt a lack of acceptance and support from the medical personnel in the hospital where their clinical training took place.

Students pointed out that especially during the pandemic, student groups should be smaller than usual so that the teacher could devote more time to individual work with every student. In addition, students emphasized the importance of paying attention to epidemiological safety principles during classes, both on campus and in healthcare facilities where they received practical training. However, they pointed to the difference between mentors from the university and from the hospital, with the former being more familiar with the procedures; they instructed the students on how they should behave around an infected person.

*“I felt more confident in classes conducted in smaller groups, e.g., in CSM (editor’s note: Medical Simulation Centre), where classes were held under the supervision of academic teachers—they took care of our safety, in contrast to what it was like in the ward. Medical staff did not always have time to share with us information on the ward’s current epidemiological status.”*

(St. 6)

#### 3.2.2. Theory–Practice Gap

According to the vast majority of students, the knowledge provided to them only in the form of theory without the possibility of taking practical classes negatively affected their nursing skills developed during their studies. Although students returned to practical in-class learning following the national lockdown, classes were often cancelled whenever a case of COVID-19 was detected in a student group or among patients/medical staff. This made it virtually impossible for the students to acquire practical skills in a clinical settings, leading to frustration and dissatisfaction. During their university education, they focused on developing and shaping new skills, but the COVID-19 pandemic delayed and slowed the progress they had made. Students would also contemplate on whether, since classes were so frequently cancelled or not scheduled at all, their graduation would be postponed.

*“At the beginning, when online classes started and our practical classes were often cancelled, I thought it would be nice to have more time for myself. However, as the time passed and we were approaching graduation, I realised there were so many things I hadn’t learned. I was annoyed when they cancelled our classes again and again because of quarantine or isolation. Obviously, I was afraid for my health and my family’s, but I really wanted to master all those practical and manual nursing skills.”*

(St. 9)

*“I felt some anxiety and uncertainty about whole education in general. I wondered if they would extend our studies and will it take me 4 or 5 years to graduate.”*

(St. 18)

Students were afraid that cancellation of classes in the event of an infection outbreak or limitations in the clinical environment that they faced during training would affect their future work, as they would have to learn practical skills from their colleagues after being employed as a nurse. In their responses, students expressed disappointment with their potential not being fully used as well as a sense of losing much time which should have been devoted to acquiring practical skills and learning how to approach patients and their families. In addition, returning to on-site classes after a country-wide lockdown posed a challenge for the students, as they did not know how patients would behave with them around, whether they would be cooperative in direct contact, or whether they would allow the students to take care of them. As stressed by the students, they had to relearn how to communicate with patients in order to know what to ask and how to behave.

*“It was frustrating because we had so few practical classes… it’s not that we didn’t have them at all, it’s just they had been reduced in number. Now I can see that this will impact the practical sphere and I will have to learn everything myself, from my fellow employees.”*

(St. 8)

*“It felt quite special to be back in class… I was also a bit afraid of how patients would react—whether they would be willing to talk or engage in any personal contact. During those few months of work in my ward, I found it increasingly easy to talk to patients in other wards.”*

(St. 2)

Students were concerned about not being able to hone their practical skills during clinical classes at the hospital under the guidance of a mentor who would supervise their performance. There were too many classes based on theory or use of phantoms, and not enough held in clinical settings. Even after classes in clinical facilities had been restored, medical staff allowed students to carry out only basic, simple nursing activities. In their accounts, the students also mentioned the methods of conducting practical classes in a situation where they could not be held in clinical settings. In their opinion, replacing practical classes with procedures or nursing processes developed for this purpose will not compensate for real-life work in a healthcare facility.

*“In the last six months of our internships we didn’t even approach or take care of patients. Instead, while in the hospital, we would sit next to the patient and write a guide, sometimes over ten-page long.”*

(St. 8)

The lack of practical classes also made the students feel disappointed. They were not sure if they could master all the skills needed to provide safe patient care. Furthermore, the first clinical classes after the lockdown were devoted to reducing the backlog resulting from the lack of practical classes. Students were more eager to participate in practical classes and internships and learn from them as much as possible, as they did not know if they would be cancelled. By participating in these classes, students could practice the nursing procedures and activities needed in everyday nurses’ work, but known to them only from theory. Students, however, emphasized their dissatisfaction with this area.

*“I think the hardest thing was to administer nursing procedures to a patient, after a few months of online and laboratory classes. Establishing contact and performing manual activities was a bit more difficult, especially if you didn’t practice them earlier.”*

(St. 20)

*“After finishing my internship, I always felt unsatisfied because we were losing so much professional learning opportunities and practical classes or we only had them remotely, so obviously we were unable to master any practical skills which are so important in this job”*

(St.1)

#### 3.2.3. Ambivalent Emotions and Ethical Challenges

Students experienced both negative and positive emotions during clinical training. They were happy when the period of exclusively remote education was over and they could return to clinical classes together with their colleagues. Some students mentioned how important it was for them to have mutual support and help from their peers in a situation where, for health reasons, someone could not participate in practical classes. At the same time, after healthcare facilities reopened for educational purposes to students of nursing, anxiety and frustration appeared among students due to the risk of contracting the virus or spreading it to their families and patients they worked with. Ethical questions had arisen; the need to enter the building and participate in classes was contrasted against the fear for patients and their relatives associated with the risk of infection. When students’ family members contracted COVID-19, they felt guilty about the possibility of being the source of this infection.

*“As for this year’s internship, when we entered the hospital I was anxious whether we would actually infect any patients or if they let us provide care to them. I also felt joy because it was possible to go out, meet the rest of our group.”*

(St. 3)

*“On the one hand, I experienced frustration and fear, thinking about my life but also of my family’s, I was afraid to participate in the classes, I lived with my dad who was a stroke survivor but I was scared for the health of my whole family. My workplace was hit with COVID and my dad got sick too. I was in quarantine. It made me feel lonely, guilty.”*

(St. 8)

Students described the various, often ambivalent emotions accompanying them. Early into the pandemic, some of them experienced loneliness due to lockdown and no contact with their peers. Feelings of isolation and loneliness would also appear when a family member got infected and the student was put in quarantine. Some students also mentioned the stress associated with a sense of chaos, i.e., not knowing what the next day will look like or whether classes will be held or cancelled again due an the outbreak of the virus in the healthcare facility or someone from their peer group being infected.

*“What I remember from the beginning of the pandemic is that I just felt lonely.”*

(St. 15)

*“I was often stressed, waking up in the morning. No one knew whether the classes would actually take place or be cancelled.”*

(St. 13)

The fear of infection also aroused emotions, especially at the early stages of the pandemic, before the COVID-19 vaccine was developed. Another source of anxiety and frustration was the information chaos regarding the procedures applicable to students in the event of contact with a patient who has tested positive for COVID-19, mainly from medical staff.

*“Probably the most difficult situation was when we were taking care of a patient only to find out on the following day that he was infected with coronavirus. We were put in quarantine, no vaccinations were available yet. I remember those 10 or 14 days as a time filled with fear, uncertainty, anticipation and similar emotions, asking myself whether I have fever or whether I will lose taste today, or do I still have the sense of taste or smell? It was at the beginning of the pandemic.”*

(St. 18)

After students returned to on-site classes in healthcare facilities, they had to take responsibility for their actions when providing care to patients. Nursing students felt that they were needed and that their tasks were important from a medical point of view. Initially, they felt fear, but gradually became accustomed to the new situation and learned how to cope with it. Concerns related to the risk of infection further decreased when vaccinations appeared. Only then did students feel that they were protected to some extent.

*“What especially caught my attention was how I behaved around patients. I tried to act responsibly to prevent any risks”*

(St. 4)

*“I felt so needed. I was responsible for the patients I had in my care and it was a very nice feeling.”*

(St. 17)

In addition, the visible loneliness of patients and lack of prospects for this situation to change increased the mental burden on students, and this was particularly prevalent during subsequent waves of the pandemic. Students noticed that older people, e.g., in residential homes, were locked for weeks in their rooms without contacting other people. It was mentally exhausting for them. Similarly, students found it difficult to cope with the loneliness of COVID-19 patients. The only people with whom those patients had any contact were members of medical staff and internship students. According to the students, nurses acted as family for patients. Witnessing how lonely patients were, students would often want to visit them in their rooms, at least for a moment.

*“All outside visits were prohibited, so the patients, especially the elderly, were really depressed. When someone from their family called, they were nervous because the staff didn’t have the time to tell them what was happening with the patient or pass on the phone so that they could talk. It wasn’t so bad for the younger patients—it was the older ones that suffered the most.”*

(St. 5)

At the same time, contact with patients was hindered not only by their isolation, but also by personal protective equipment, e.g., masks, which make communication difficult. The ban on visits evoked a sense of loneliness in patients, but also drew the staff’s attention to them without the need to contact their families.

*“Patients would often tell us they could not understand what we were saying and that they would prefer talking without the mask. They found it difficult to understand anything and grasp the information we wanted to share (…) Currently, all our attention is devoted to the patient only, whereas before the pandemic there was also the family. It was quite different then and to be honest I’m a little worried about how I’ll handle contacting both the patient and his or her family.”*

(St. 3)

Some of the most difficult experiences reported by students during their hospital internships included the first encounter with a patient connected to a ventilator, resuscitation, the witnessing of death, and assisting in the provision of care to terminal patients in critical condition. Students readily admit that they are not mentally or emotionally prepared for such situations. Equally stressful for them was the task of working with aggressive patients and witnessing for the first time the use of direct coercive measures. Another difficult situation recalled by some of the nursing students was noticing that the patients they had been caring for died.

*“What mostly stuck in my mind was cardiology classes, where for the first time ever I witnessed resuscitation and death of a patient. (…) As I said earlier, this was the first time I saw someone die in the ward; the nurses asked us to help with post-mortem care, but I didn’t feel quite ready yet.*

(St. 13)

*“During my internship at the internal medicine ward, I had my first case of death, which was also the first event of this kind in my life. (…) For me, the most difficult and stressful situation was to care for a patient in a state of drug starvation—he was being aggressive, sedatives did not work on him, and he tried to escape from the ward (…) also, there was the risk of contracting HIV or some other virus. Another time three people died in the ward in just four days—two of these deaths were discovered by us, the students.*

(St. 1)

During clinical training, in addition to fear, nursing students were also accompanied by a sense of helplessness with regard to patients whom they could no longer help in any way. The students were experiencing physical (due to the multitude of classes and responsibilities) and mental exhaustion. This made it even more difficult for them to concentrate in class and acquire new skills. As one student recalls, she could only cope with this unusual and difficult situation in the midst of the pandemic owing to her composure and inner calm.

*“What stuck in my memory the most? I think I’ve become so desensitised that nothing moves me anymore. (…) When we had classes at a care and treatment facility, I was struck by my helplessness towards those patients, (…) there was nothing more we could do for them (…). In addition to fatigue, both physical and mental, there were also nurses who didn’t always want to teach us everything. We had to use phantoms and they would tell us to “take temperatures” or “check blood pressures” rather than give us any real-life assignments. The nurses did not really let us experience what their work was about, they would rather see us as a sort of care assistants. In all this, I was being helped by my inner and composure.*

(St. 7)

The students also noted that even though patient communication forms an important part of the curriculum in nursing studies, some nurses on the ward hardly ever talked to the patients. The second group of respondents, on the contrary, stated that only nurses held actual conversations with the patients, especially with those infected with the SARS-CoV-2 virus.

*“I am (…) disappointed with nurses, (…) I know I’m still young, but sometimes I feel that this nursing role model that I had imagined got lost somewhere. Especially when I witnessed nurses avoiding contact with patients. I had my health concerns too and I worried about my family’s safety, but we all knew what this profession entailed when choosing it as a career”*

(St. 11)

#### 3.2.4. To Be Part of the Team

Another category which had been distinguished is to be part of the team, the peer group, therapeutic team, or nursing team. The students stated that the relationships within the study group helped them to a great extent to accept and cope with the COVID-19 pandemic. They were there to support each other, share advice, and offer help to fellow students. Whenever the situation called for it, they would cheer each other up to ease the stress and tension. They also trusted each other and exchanged information, e.g., when coming into contact with a SARS-CoV-2 patient or regarding their health status and any worrying symptoms.

*“As for us, students, we supported and trusted each other very much, we offered help whenever someone could not participate in the training or classes.”*

(St. 14)

During clinical training, they helped each other and ensured that everyone had an equal opportunity to perform and practice the nursing activities they had been learning about. During classes held online, however, contacts between students remained at the same level or were slightly reduced, so that actual relationship building was only possible after return to on-site learning.

*“When it comes to personal relationships, nothing really changed in my peer group from the time before the pandemic. I’d say that they even improved upon our return to school following the suspension of classes in early 2020… we made sure that everyone had the opportunity to try out all of the ward procedures (…)”.*

(St. 2)

*“As regards our student group, I can say we all really trusted each other. Whenever anyone had some health concern, he or she would inform the rest.”*

(St. 6)

Students also emphasized that they wanted to be part of the therapeutic team. They did not want the hospital nurses to treat them as someone who is unwelcome, an intruder who only interrupts their work and hangs around aimlessly. The students felt that they were not being treated on equal terms and experienced a lack of respect from medical staff. They also often found themselves uncared for and unsupervised by the head nurse, with the consequence of being assigned to random nurses who were not always interested in or willing to act as their teachers.

*“As regards our supervisor at the hospital…, I had an unpleasant experience with information being withheld from us. I believe it was due to the nurses not treating us as equal members of the therapeutic team. I remember a situation when they didn’t tell us that the patient we were taking care of was infected with coronavirus… this really confused us and we didn’t really know how to proceed from there.”*

(St. 14)

The students declared their willingness to be part of the nurses’ work. Unfortunately, they would be pushed away from the patients and assigned non-nursing tasks. Students complained about this, first approaching the personnel of healthcare facilities in which they had their internships. They would then mention that, in accordance with what they had been taught during ethics classes, it is the duty of a senior nurse to serve as a mentor to her less-experienced colleague, something that the students missed. In their experience, nurses who worked in hospital wards believed that educating students was the sole responsibility of a dedicated mentor such as the head nurse.

*(…) I can’t say I’m happy with our supervisor, the head nurse. I didn’t feel welcome in the ward, We were being pushed away from the patients and at beck and call of the staff, if anything.*

(St. 11)

*“For me, it is always sad to realise that nobody actually wants to teach you anything and that you feel like a fifth wheel. And that’s regardless whether with or without a pandemic.”*

(St. 10)

During clinical classes, the respondents could witness different behaviors of nursing staff. Some of them were disappointed with the behavior of nurses they worked with during clinical classes, i.e., their attitude and lack of commitment. Others, in contrast, had fond memories of these classes and appreciated the commitment of the ward nurses. The students concluded that workflow in a therapeutic team was smoother and more efficient when they were adequately instructed and guided beforehand by the staff and university teachers with regard to which orders and nursing activities they could perform.

*“I am disappointed. Disappointed with nurses, I had unpleasant situations with some of them, I know that I am young, but sometimes I have the impression that the nurse role model does not exist.”*

(St. 11)

*“Those experiences varied—some of those people were nice, while others not so much. Some were afraid of COVID, while others just ignored it.”*

(St. 12)

#### 3.2.5. Strengthened Professional Identity

The experience of participating in clinical activities during the COVID-19 pandemic reassured some of the students about their choice of career. Despite the many adversities associated with remote learning (the number of assignments to be completed, partial or total unavailability of in-person contact), it has not altered students’ attitudes towards their profession.

*“It was especially during the remote classes that I felt displaced, outside the path I had chosen—nursing. My impression was that I was moved away from the patient, the hospital, and the healthcare system as a whole, while being mentally burdened by the millions of papers I had to write. The moment I returned to clinical classes I was very excited and this experience made me even more convinced about nursing as my future career.”*

(St. 12)

At the same time, however, students pointed out the time pressure they were under when performing their assignments and the concerns associated with there being no foreseeable end to the pandemic. Despite these setbacks and the fact that work placement took place during the pandemic, students were still willing to undertake employment in the nursing profession.

*“I still want to be a nurse, but I’m afraid these pandemic waves will never end.”*

(St. 7)

The difficult experiences associated with the COVID-19 pandemic have emphasized in the nursing students the need to provide help to anyone in need. Regardless of the many difficulties (the evolving pandemic restrictions, the requirement to use protective suits at work, and the fear of contracting the coronavirus), the respondents appreciated the opportunity to come into contact with patients for longer periods of time and also felt proud being able to test in real-life conditions the competencies they had acquired so far and to verify their theoretical knowledge.

*“I believe this is a different, definitely better experience, because I get to do things I enjoy and have more contact with patients, as compared to standard work placements in a hospital ward. I also appreciate the fact that during such a short period I was able to learn so much and try to be a professional nurse.”*

(St. 2)

*“I was only hoping that the attitude of the current government towards healthcare sector would change—but, over time, they have disappointed me with what they do and how they act towards us.”*

(St. 6)

Students stressed that their attitude towards the nursing profession had not changed. By experiencing various situations during clinical classes, they realized what kind of nurses they would like to be and how they can approach patients and their families. They also noted that nursing is a dynamically developing profession with many challenges and unexpected situations to be prepared for.

*“This experience has certainly helped me realise what kind of nurse I want to be.”*

(St. 10)

The surveyed students also noticed that their university studies and hospital work placements were fundamental for their nursing careers. It is a stage when they take up a particular direction, broadening knowledge in a specific field, coming into contact with patients for the first time, and learning new practical skills. The time at university is a kind of protective period, as students have an academic teacher to guide them and correct their mistakes if necessary. However, many of them realized that the right to practice does not guarantee that they will have trust in their competencies as nurses. They are aware of the need for continuous self-learning, which they believe is the best way to master a profession after graduating from university.

*“Now I see it in a bit different light. At first I thought that someone would teach me, that I would learn the ins and outs of this profession at the university or hospital. But apparently I will have to put in a lot of my own time learning about it, even after I get my license. That doesn’t mean I’ll be able to do everything.”*

(St. 17)

The students also noted that nursing is a difficult profession which has its specific features. It requires great dedication, not only in terms of the need for lifelong learning, but also due to limited time for family and friends. The foundation of nursing work is another person, with the relevant fact being that this person is afflicted by a disease; the ability to establish contact therefore forms an essential part of every nurse’s work.

Another aspect emphasized in interviews was the role of the nurse in a health facility. The nurse takes an active part in the therapeutic process, cooperating with the physician and helping to develop a bond between them and the patient. According to the students, nursing staff are perceived by patients and their families as a reliable source of information as well as trustworthy professionals who provide support to those in need. Notably, the level of a patient’s trust towards medical staff in general is largely dependent on nurses’ conduct.

*“I noticed that families saw nurses as an important source of information and support for the hospitalised patients.”*

(St. 20)

## 4. Discussion

The aim of this study was to explore the perceptions of nursing students of their practical training in the period of the COVID-19 pandemic. The study was based on an analysis of interviews with nursing students (BA and MA degrees) who took part in practical training in different clinical areas in the years 2020–2021. Five main categories emerged: (1) the key role of a clinical mentor; (2) theory–practice gap; (3) ambivalent emotions and ethical challenges; (4) to be part of the team; and (5) strengthened professional identity. The COVID-19 pandemic has magnified the problems faced by nursing students during clinical classes, presenting them from a new and hitherto unknown perspective. The emerged categories reflect the main problems faced by nursing students during any clinical training documented in many studies [10,11,12,13,14,15,16,17,18,19].

The first featured category, ‘the key role of clinical mentor’ depicting the key role of clinical mentor in nursing training, as has been emphasized for many years in the literature [36,37,38,39,40]. The pandemic has stressed the irreplaceable role of the mentor in clinical training with respect to teaching future generations of nurses and midwives. Respondents said that clinical mentors tried to teach them theoretical and practical knowledge at the highest level, so that students in their future professional work could provide high-quality healthcare. As the research shows, collaboration between a student and a mentor during clinical training, and especially the time devoted to the student, helps students gain confidence and improve their practical nursing skills [41,42]. In addition, when working with mentors, students feel their support, which increases their sense of security while performing nursing activities [37,38]. Therefore, an effective relationship between a mentor and a nursing student contributes to the professional development of the student [43].

Students mentioned various mentor–student relationships. Some of them experienced a lack of acceptance and support from the medical professionals working in the hospital. The reasons for this behavior of medical personnel can be found in the study by Soto-Rubio et al. [44], where the authors point to the role of emotional intelligence, which is significantly burdened by high levels of stress, generating many unpleasant situations in the workplace. Additionally, it was important for teachers to protect students’ physical and mental health as well as show support and understanding. In both Fitzgerald et al. [45] and our own study, nursing students who received support from an academic instructor manifested less anxiety in response to difficulties resulting from the COVID-19 pandemic. Good communication with students, patience, and willingness to teach, i.e., aspects characterizing academic teachers, contributed to an improved learning environment. Students would also find it important if teachers devoted sufficient time to explain to them incomprehensible information and difficult clinical procedures [45]. Furthermore, the study by Kaihlanen et al. [39] shows that clinical mentors have an impact not only on students’ motivation to learn, but also on their ability to adapt to new, difficult situations, with the mentor being considered as an example to follow in future professional practice.

The next category analyzed, ‘theory–practice gap’, highlights, among other things, respondents’ concerns about the possible cancellation of clinical classes. As reported by Saifan et al. [46], theoretical knowledge derived from the latest research findings serves as the basis of nursing practice; however, it is the environment in which clinical activities are carried out that determines how knowledge is applied in practice. Transferring theoretical knowledge into clinical practice is only one of the challenges faced every day by nursing students and their clinical supervisors, but also by licensed nurses at the early stages of their careers [46]. Nursing students felt disappointed about the absence of practical activities because they assumed this could affect their future careers. Studies show that the gap between theory and practice may be related to both medication preparation errors [47] and satisfaction with the profession [48]. Lack of preparation for clinical work was also reported by students in the study by Collado-Boira et al. [17]. The respondents were concerned about the possibility of making a medical error, and emphasized their inability to make quick decisions in difficult clinical situations, even though they felt that practical classes forming part of their curriculum had prepared them for work [17]. They were not sure if they could master all the skills needed to provide safe patient care. Similarly to our own study, Casafont et al. [19] reported that for students, the practical activities during the COVID-19 pandemic were a very important and valuable experience because of the skills they learned. Clinical activities taking place during the pandemic allowed students to practice the nursing procedures and activities needed in their daily work, as well as learn how to use modern medical equipment. Strategies that can assist in bridging the gap between theory and practice in the education of nurses and midwives are the clinical scenario management standards available to staff [49].

Another important category is the one relating to students’ conflicting emotions and ethical dilemmas in clinical practice. In both the aforementioned study and our own study, students expressed concerns about being potential carriers of the COVID-19 virus among their relatives and colleagues [17]. The findings from this study are consistent with the results obtained by Collado-Boir et al. [17], where the respondents (*n* = 65 nursing students, *n* = 82 medicine students) reported concerns about inadequate COVID-19 protection measures when providing care to patients. Research confirms that the stress caused by clinical classes is greater than during theoretical classes, negatively affecting both students’ daily lives and their educational achievements. Moreover, one of the most stressful situations in nurse education is the first clinical training [17]. When asked about their emotions, respondents expressed conflicting views. Some of them, at the beginning of the pandemic, felt lonely and stressed, not knowing what to expect of the following days, i.e., whether classes would proceed as planned or be cancelled again. The same conclusion was reached by Dewart et al. [50], who highlighted students’ concerns regarding the reduced number of practical activities. Other participants, however, expressed joy whenever classes were cancelled so that they could go out and meet their peers from the student group. Interestingly, more than half of the students participating in the study by Carmignola et al. [51] welcomed the possibility of adapting learning activities to their daily schedule. Haslam [52] points out that online learning may fit well into their lifestyles, work, and household chores.

Literature on the subject reports that nursing students are at risk of higher stress levels as compared to students of other academic disciplines [53]. Among the most difficult situations encountered by the respondents as part of their work placements included care of a terminal patient who required a ventilator, participating in resuscitation, and witnessing death. Students readily admit that they are not mentally or emotionally prepared for such situations. Ethical problems experienced by nurses in the course of their work require the ability to recognize them and to develop adequate coping strategies [54]. Indeed, stress, anxiety and the inability to cope may result from insufficient preparation of students for classes in a clinical setting [55]. According to Sand [55], students lack the skills necessary to provide care to patients with multiple conditions, which in turn contributes to low self-esteem regarding one’s own nursing skills.

Another category which had been distinguished is belonging to a team (to a student group or a therapeutic team). The students stated that the relationships within the study group helped them to a great extent to accept and cope with the COVID-19 pandemic. Sand [55] suggests that in order to reduce anxiety and stress and improve the sense of well-being, faculties should pay particular attention to promoting healthy lifestyles as well as exercising a supportive, caring and empathetic attitude towards students, faculty, colleagues, and themselves. These measures, applied in difficult times, can help students remain focused and adapt to new conditions [55].

The study also revealed the difficulties of cooperation between students in clinical practice and nurses employed in wards during the COVID-19 pandemic. Respondents described their participation in practical patient care as interfering with nurses’ work. These findings are consistent with previous studies [10,18,56], in which students felt they were a burden to nurses due to not being assigned any clearly defined roles or tasks. The feeling of being ignored by nursing staff during practical sessions accompanied both the students who took part in our study and those in the study by Rodríguez-Almagro et al. [57]. Respondents pointed to issues such as the inability to ask questions to nurses and not being given enough attention or having their performance supervised; these sentiments were also reflected in our study [10,18,56]. Difficulties in establishing a sense of mutual cooperation between a student and a nurse within a therapeutic team may be associated with problems experienced by nurses working in a hospital during the COVID-19 pandemic, such as stress, emotional exhaustion, anxiety or depression, leading to lower caring capacity and poorer quality of patient care [58,59]. Hence, it could be beneficial to conduct a study on this matter.

Nursing students also noticed some changes (e.g., regarding professional responsibility) in the daily work of nurses during the pandemic. These resulted from the introduction of new and the adjustment of existing procedures and standards of practice. This corresponds with the findings of Galletta et al. [60], who report increasing requirements associated with nurses’ work and organizational skills. Furthermore, almost all participants in the study found work placement during the COVID-19 pandemic immensely satisfying on both personal and professional levels. Similar study results were observed by Rodríguez-Almagro et al. [57]; work placements organized during the pandemic made students realize that the career they had chosen was not accidental and that they want to further their education, bringing help to all those in need. Many of them felt like a “full-fledged” part of the healthcare system, i.e., they experienced independence, but also responsibility for the health and lives of the patients under their care.

The last featured category, ‘strengthened professional identity’, showed that the experience gained by the respondents during clinical placements in pandemic conditions resulted in a stronger conviction that nursing is the right career choice. The building of nurse’s professional identity starts from admission to university and is followed by acquisition of theoretical and practical knowledge during clinical classes, and, eventually, professional work [61]. By participating in classes in the specific pandemic conditions, the students could witness the nursing qualities they would like to replicate in their future careers or, in case of witnessing unprofessional behavior, they would like to avoid in favor of better nursing practices. Students’ accounts in the study by Swift et al. [62] revealed that the pandemic and attitudes of the general public towards nursing gave them further reassurance at the start of their professional journey; when set against the context of the pandemic, they perceived their profession as a calling. It is proven that nursing students with lower professional identity more frequently plan to leave the nursing profession [63]. It is also believed that the COVID-19 pandemic had a positive effect on the image of the nurse perceived by the nursing students. It could be a result of the role played by the nurses, their responsibility, and increasing professional prestige during a pandemic which showed students important values of the nursing profession [63].

### Study Limitations

The study presented here has several limitations. First, it is a qualitative study that addresses a narrow group of respondents. At the same time, however, the findings are not intended to generalize, but to signal and explore how nursing students understood their perceived reality during the COVID-19 pandemic with regard to practical preparation for nursing work. Hence, it would be advisable to carry out similar studies in other contexts in order to obtain a more thorough understanding of this issue.

## 5. Conclusions

The pandemic has brought about many changes in the functioning of healthcare and the education of nurses. It has also underlined the role of mentors in education, providing the opportunity to develop new strategies for introducing students to clinical practice based on current medical knowledge and modern technologies (medical simulation, telemedicine, virtual reality) and ultimately to mitigate the gap between theory and practice and reduce the stress and anxiety associated with inadequate preparation for professional work. The analysis of the results also encourages the introduction of clinical teaching control measures and the improvement of mentoring schemes. Free psychological support and training courses on strategies for coping with stress and anxiety, ethical problem solving, and decision-making should be available to every nursing student. The results of our research indicate that higher education institutions should implement clear strategies to help students, both in terms of psychological support and compensation of knowledge and professional skills, the development of which might be limited during the pandemic.

## Figures and Tables

**Table 1:** Characteristics of first- and second-cycle studies in the field of nursing [32,33,34].

	First-Cycle Studies (BA)	Second-Cycle Studies (MA)
Duration of university education	min. 6 semesters	min. 4 semesters
Number of hours of classes	min. 4720 h	min. 1300 h
ECTS (European Credit Transfer System) score	min. 180 h	min. 120 h
Groups of classes in which detailed learning outcomes are achieved	basic: 500 h, social and humanistic: 420 h,	social and humanistic: 270 h, advanced nursing practice: 510 h,
	basic nursing care: 600 h,	scientific research and development of nursing: 170 h
	specialist care: 900 h	
Practical Classes		
Number of hours	1100 h	Not applicable
Example of practical classes carried out	pediatrics and pediatric nursing: 160 h internal diseases and internal medicine nursing: 120 h primary healthcare: 120 h	
Person in charge	academic teacher, other authorized persons having the right to practice the profession of nurse or midwife, min. one year’s professional record in the field corresponding to the classes conducted	
Professional Internships		
Number of hours	1200 h	200 h
Example of professional internships carried out	fundamentals of nursing: 120 h	oncological care: 40 h
	primary healthcare: 160 h	endoscopy laboratory: 40 h
	anesthesiology and nursing in life-threatening situations: 80 h	management in nursing: 20 h
Person in charge	an employee of a healthcare facility offering professional internships to students, having the right to practice the profession of nurse or midwife	

**Table 2:** Questions which guided the interview with students.

Questions
1. Can you talk about the practical classes and the internship you took during the pandemic in a clinical setting that stuck in your mind the most? What made you remember these classes the most? What was special about them?
2. What were your feelings during practical classes and internship in a clinical setting during the pandemic?
3. Do you remember the relationships within the therapeutic team and in your peer group; did anything draw your attention in particular? Can you tell me about it?
4. Do you remember your contact with patients/their families; did anything draw your attention in particular? Can you tell me about it?
5. Do you remember your contact with the mentor/supervisor of practical classes; did anything draw your attention in particular? Can you tell me about it?
6. Do you remember any particularly difficult situation that you had to face during practical classes/internship in a clinical setting during the pandemic? Can you describe it?
7. What aided/facilitated your participation in practical classes/internship in a clinical setting and the completion of nursing tasks during the pandemic? And what made it difficult to participate in these classes?
8. How would you describe your attitude towards your professional work now; do you think it is different than it was before the pandemic?

**Table 3:** Socio-demographic characteristics of participants.

Socio-Demographic Characteristics		M	SD
Age		22.15	1.19
		*n*	%
Gender	Women	17	85
	Men	3	15
Studies	Bachelor’s degree 1st level	15	75
	Master’s degree 2nd level	5	25
Place of residence	Urban	12	60
	Rural	8	40

M—mean, SD—standard deviation.

## Data Availability

The data presented in this study are available upon request from the corresponding author.

## Supplementary Materials

The following supporting information can be downloaded at: https://www.mdpi.com/article/10.3390/ijerph19106352/s1, File S1. COREQ (Consolidated criteria for Reporting Qualitative research) Checklist.
